# A Brief Survey on No-Reference Image Quality Assessment Methods for Magnetic Resonance Images

**DOI:** 10.3390/jimaging8060160

**Published:** 2022-06-04

**Authors:** Igor Stępień, Mariusz Oszust

**Affiliations:** 1 Doctoral School of Engineering and Technical Sciences, Rzeszow University of Technology, al. Powstancow Warszawy 12, 35-959 Rzeszow, Poland; igorkrzysztofstepien@gmail.com; 2 Department of Computer and Control Engineering, Rzeszow University of Technology, Wincentego Pola 2, 35-959 Rzeszow, Poland

**Keywords:** survey, image quality assessment, no-reference image quality assessment, magnetic resonance images

## Abstract

No-reference image quality assessment (NR-IQA) methods automatically and objectively predict the perceptual quality of images without access to a reference image. Therefore, due to the lack of pristine images in most medical image acquisition systems, they play a major role in supporting the examination of resulting images and may affect subsequent treatment. Their usage is particularly important in magnetic resonance imaging (MRI) characterized by long acquisition times and a variety of factors that influence the quality of images. In this work, a survey covering recently introduced NR-IQA methods for the assessment of MR images is presented. First, typical distortions are reviewed and then popular NR methods are characterized, taking into account the way in which they describe MR images and create quality models for prediction. The survey also includes protocols used to evaluate the methods and popular benchmark databases. Finally, emerging challenges are outlined along with an indication of the trends towards creating accurate image prediction models.

## 1. Introduction

The advantages of magnetic resonance imaging (MRI) over other medical imaging methods, including computed tomography (CT), X-ray and ultrasound imaging, and positron emission tomography (PET) are based on its safety and ability to provide detailed images in axial, sagittal, and coronal planes [1]. Delving into the subject of improving the MRI quality, attention should be paid to the characteristics of the researched material. Protons that are normally and randomly oriented within the water nuclei of the examined tissue are arranged by a powerful, uniform, and external magnetic field. The most commonly used MRI sequences are T1-weighted and T2-weighted scans. T1 (longitudinal relaxation time) is a fixed time that determines the velocity at which excited protons return to equilibrium, while T2 (transverse relaxation time) is the constant that determines the rate at which excited protons leave the phase with each other or reach equilibrium. T1-weighted images are produced with the use of a short time to echo (TE) and repetition time (TR). Its properties determine the contrast and brightness of the image. On the other hand, the T2-weighted images are produced by using longer TE and TR. Another broadly used sequence is the fluid-attenuated inversion recovery (FLAIR). This sequence is similar to T2-weighted images aside from the fact that the TE and TR times are much longer. It is exceptionally sensitive to pathology and facilitates the differentiation between cerebrospinal fluid and an abnormality [2,3]. MR imaging is prone to distortions due to many reasons such as signal or image operations, equipment characteristics, or operator mistakes [4]. Examples of operations that cause distortions are acquisition, enhancement, compression, or reconstruction procedures. Furthermore, unexpected patient movement or an error made by the operator performing the test can introduce additional and unwanted visual information to acquired images. These factors have an impact on the subjective assessment of the obtained image and the subsequent diagnosis. The scanners are used to provide a sequence of images valuable for further diagnostic purposes. They are created with varying magnetic field strength and measured in Tesla units (T). Nowadays, the most commonly utilized magnet strengths in MRI machines are 1.5 T and 3 T. The 1.5 T scanner can be considered as commonly used in clinical settings, whereas 3 T is then employed in research settings. Comparing two devices, some implants that are safe to go into a 1.5 T scanner may not be safe for the patient in a 3 T scanner. On the other hand, 3 T scanner provides better image quality and faster scan speeds [5]. Therefore, automatic and objective image quality assessment (IQA) is particularly important. With the growing development of IQA research, several new algorithms have been created. This paper aimed to briefly elaborate on the MR-IQA methods suitable for the evaluation of MR images. To the best of the authors’ knowledge, such methods have not been covered in a single survey since related surveys describe the general methods for IQA or methods designed for medical images with the focus on fetal brain MRI [6,7]. Furthermore, since their creation, new MR IQA methods have emerged.

The assessment performed by the method should be as close as possible to the subjective assessment, and with the development of reliable techniques, the human assessment in MRI may be minimized or, in the longer term, completely replaced. The objective approaches for IQA can be categorized into full reference (FR), reduced reference (RR), and no reference (NR)/blind image quality (BIQA) methods [8,9,10,11]. In the case of FR and RR, an undistorted reference image, or a part of an image to which the assessed image is compared, should be available. However, in the case of medical imaging, such an undistorted image does not exist [12]. Therefore, the development of BIQA methods is of particular importance in this field [13,14,15,16]. NR methods can be further classified into opinion-aware and opinion-unaware/completely blind approaches [17] depending on the access to subjective scores while creating a quality model.

The rest of the paper is organized as follows. In Section 2, selected MRI distortions are described. Section 3 presents NR-IQA features and briefly characterizes MRI methods. Section 4 introduces databases and MRI NR-IQA protocols. Finally, Section 5 summarizes the achievements in NR-IQA of MR images and indicates the main challenges and future directions.

## 2. MRI Distortions

**Figure 1:**
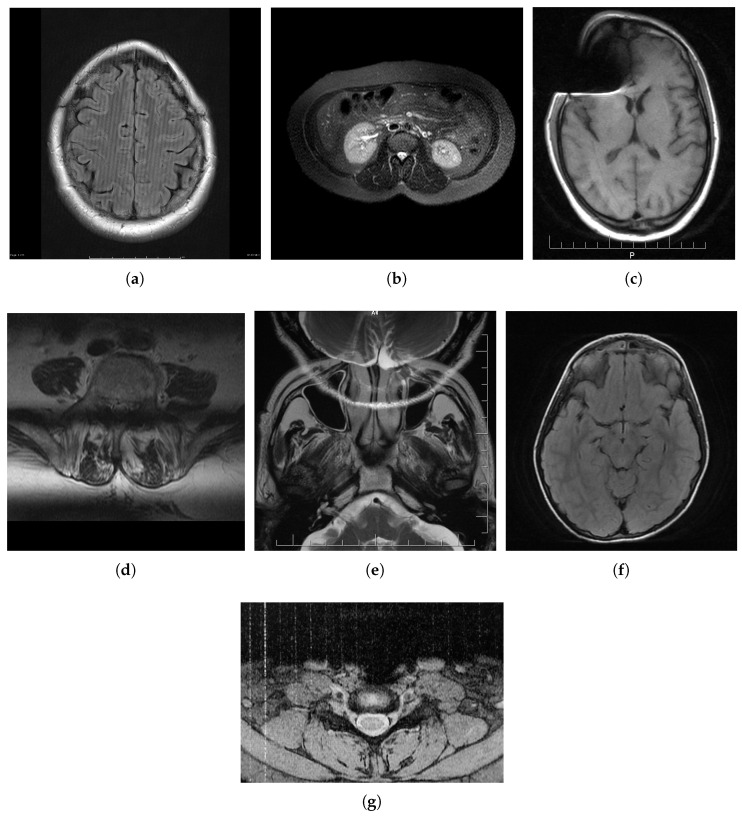
Examples of artifacts in MR images: (**a**) herringbone; (**b**) ghosting; (**c**) magnetic susceptibility; (**d**) slice overlap; (**e**) aliasing; (**f**) Gibbs effect; and (**g**) zipper. Cases were the courtesy of: (**a**) Assoc. Prof. Frank Gaillard, rID: 19695; (**b**) Assis. Prof. Faeze Salahshour, rID: 81727; (**c**) Dr. Ayush Goel, rID: 22731; (**d**) Dr. Roberto Schubert, rID: 16705; (**e**) Dr. Prashant Mudgal, rID: 26927; (**f**) Dr. Prashant Mudgal, rID: 27302; and (**g**) Dr. Alan Nazerian, rID: 45665; radiopaedia.org (accessed on 27 April 2022).

Here are several categories of distortions, sequence artifacts, or distortions related to image reconstruction that can be minimized by using an appropriate work protocol, system improvements, or enhancing image quality improvement and processing methods. Moreover, there are system artifacts due to the misused or faulty MRI equipment. However, sample artifacts or human-induced distortions are more complex as their elimination requires not only a thorough understanding of the patient’s anatomy and psychology but also the use of specific pulse sequences [18]. The most common distortions are briefly described below and their examples are shown in Figure 1.

### 2.1. Spike (Herringbone) Artifact

The noise spike points in the k-space are generated by gradients that have been used in a very high duty cycle. The k-space is an extension of the Fourier concept, defined as an array of the numbers representing spatial frequencies in two or three dimensions of an object [19,20]. The distortions in the k-space are manifested by single or multiple points varying in intensity compared to their surroundings. The combination of information during the Fourier transform with the spike causes dark bands to be superimposed on the image. The direction, angle, and distance between the bands depend on the displacement of the noise spike from the center of the k-space. Spike noise usually occurs from loose electrical connections and is more noticeable when using high-duty-cycle sequences. In this case, following the changes in the position of the pattern produced by the spike can be used in further diagnosis [21,22].

### 2.2. Zipper Artifact

A zipper artifact is an area of alternating light and dark pixels. This distortion is placed in the direction of the frequency coding and appears throughout the series of images. It is caused by the leakage of electromagnetic energy into the magnet room or by electronic equipment brought into the room and a breach of RF shielding in this equipment. Another source of the zipper artifact is an external light source reaching the receiver coil as a result of an open door in the room where the scanner is located [23,24].

### 2.3. Ghosting

Images are influenced not only by the physical reaction of the patient during the examination or environmental factors but also by the pulsating movement. For example, the ghosting artifact can be caused by cardiac beats, arterial or cerebrospinal fluid (CSF) pulsations, respiration, and implants. When the motion is strong enough, the distortions, resembling the shape of the imaged organ, overlap with the image itself. The arrangement may resemble parallel imaging artifacts whilst resembling clots or dissections. Parallel imaging is a method of improving MRI data acquisition that works by obtaining a reduced amount of k-space data with a series of receiver coils [25,26]. The distance between the distortions may depend on the frequency of movements and the repetition time. This artifact is also categorized as motion distortion [27,28].

### 2.4. Blurring

Blur is a motion-type distortion that occurs as unevenly distributed over the entire area of an image. It has a large impact on the strength of edge visibility, weakens the clarity of the image, and reduces the contrast between different anatomical structures [29,30].

### 2.5. Aliasing Artifacts

Aliasing artifacts appear when the field of view is smaller than the body part being imaged. The field of view is an area designated by the size of two- or three-dimensional spatial encoding space of the MR image [31]. Aliasing occurs in the direction of phase coding or cross-section in 3D acquisition. `The moire’ or `fringe artifact` is a type of distortion that arises in the frontal lobe, where large fields of view produce these distortions. One way of reducing or aligning the distortion is to change the imaging axis so that the part of the body under study fits within the field of view. However, this can result in other types of artifacts. An alternative approach is to increase the field of view phase or apply spatial saturation pulses outside the field [32,33].

### 2.6. Gibbs Effect

Gibbs artifacts—also known as truncation artifacts or ringing artifacts—are a series of lines in the MR image that appear parallel to the area in which there has been a sudden and intense change in signal intensity. The Gibbs ring distortion is also mainly produced due to insufficient samples in the direction of phase coding or reading [34].

### 2.7. Slice-Overlap Artifact

The slice-overlap artifact is associated with the loss of the signal visible in the image from the multi-angle acquisition. With this distortion, the edge sections have a reduced signal intensity and do not create a section profile with a straight edge. To reduce the effect of the artifact, the angle of intersection between the ply groups should be decreased. If there are difficulties in the performed examination, e.g., damage occurs between the layers, the method of interleaving is used [35].

### 2.8. Gradient-Related Distortion

This distortion is characterized by image compression and the inadequate rebound of spins on the edges of images. It is created when the electric current receives the voltage from the gradient coil while tapering to the sides of the magnet [36,37].

### 2.9. Parallel Imaging Artifact

In general, parallel imaging is a widely used method for accelerating the acquisition of MRI data in which the distribution and sensitivity of the receiver coils play a major role. This has its positive results in reducing the imaging time but may cause distortions. As a consequence of the parallel imaging, in which each coil is at a different distance from the pixel, the signal recorded by such a coil changes and the closer coils have stronger signals. Professionals analyzing the MR images obtained from such an operation can misdiagnose the approximate source of the MR signal. In parallel imaging, the signals from the individual coils are amplified and simultaneously processed along separate channels, keeping the identity to the end. Furthermore, it may suffer from inhomogeneous noise in reconstruction and unevenly distributed noise caused by the overlapping of different structures whilst lacking the core information [26,38].

### 2.10. Susceptibility Effect

By placement in a magnetic field, the tissues become temporarily magnetized. However, magnetization is not uniform and depends on the magnetic susceptibility of the tissue, as in the case of the air–tissue or bone–soft tissue interfaces. Bone and air are less magnetically susceptible which means that a low-intensity signal is generated at these sites. Such local variations in intensity give rise to geometric distortions in the images. Another problem, also causing the non-uniformity in the signal, is a magnetized implanted device. Metal implants have a much higher magnetic susceptibility than the rest of the tissues of the human body, leading to signal distortions related to higher signal intensity [2,39].

## 3. NR-IQA Approaches

NR-IQA approaches are used in cases in which a reference image is not available. Hence, such approaches support examination based on MR images by rejecting scans of unsuitable quality. There are NR-IQA methods for medical images taking into account distortions, e.g., noise, compression, or blur. For example, based upon the human visual system (HVS) [40,41], Bhateja et al. [42] used two-stage MRI fusion metrics for IQA, where two images are fused to improve the detection of distortion. With the objective of developing automatic deep learning methods, Xu et al. [7] introduced a semi-supervised technique devoted to the IQA of fetal brain MR images with the use of a mean teacher method and a region-of-interest (ROI) consistency. Furthermore, Liu et al. [43] used semi-supervised learning to solve the problem of creating noisy annotation in the image segmentation task. This three-staged quality assessment technique employs a hierarchical residual model, and as the consequence, provides an assessment of the slice, volume, and subject level. Another classification method uses unpaired generative adversarial network (GAN) and weakly supervised trained classifier to assess MR images [44]. To address the problem of wasting potentially important 3D spatial information, the HyS-net approach was created. It was based on a hyper-network and is capable of self-adaptation [45]. A more recent approach adapting the modified blind/referenceless image spatial quality evaluator (BRISQUE) was proposed by Chow and Rajagopal [46] in which BRISQUE [47] aims to quantify possible losses of `naturalness` in the image using the scene statistics of mean subtracted contrast normalized (MSCN) coefficients.

Table 1 provides a brief comparison of NR-IQA approaches devoted to MR images. Several methods focus on specific artifacts, e.g., QEMDIM [48], or an approach introduced by Nabavi et al. [49]. However, most methods are designed to assess overall image quality considering the characteristics of MR images: modified-BRISQUE [46], R50GR18 [50], ENMIQA [51] or NOMRIQA [52]. The number of features depends on the way that the images are described from simple entropy to features extracted from the layers of a neural network. Most approaches were validated on one or two databases, among which large datasets that are assessed by more than several specialists are rare. A more detailed presentation of the methods is provided below. An experimental evaluation of recent approaches and related discussion on their performance can be found in [50] or [45].

### 3.1. A Two-Step Automated Quality Assessment for Liver MR Images Based on Convolutional Neural Network

In the method proposed by Wang et al. [53], a two-step method applied for image classification purposes was used. The method focuses on the regions of interest (ROI), i.e., each patch assessed by the radiologists as diagnostic or non-diagnostic is used to train a convolutional neural network (CNN) for segmentation purposes. Then, another CNN is used to classify the quality of extracted patches. Overall image quality is assessed on the basis of the number of non-diagnostic patches in all the liver patches of the image.

### 3.2. Semi-Supervised Learning for Fetal Brain MRI Quality Assessment with ROI Consistency

Semi-supervised learning for fetal brain MRI quality assessment with ROI consistency is a semi-supervised deep learning method that responds to the difficulties arising in fetal MRI [7]. The method is based on the average teacher model to control the consistency between the student–teacher approach, thanks to the aggregation of network parameters at different stages of training. It uses MR images classified according to the following criteria: diagnostic, non-diagnostic, and images without a brain region of interest. In this method, improved accuracy in detecting the non-diagnostic images of the brain’s ROI during feature extraction is obtained. Importantly, the method is introduced and implemented on the MR scanner, which makes it possible to check the condition of the obtained image and if necessary, repeat the examination.

### 3.3. No-Reference Image Quality Assessment of T2-Weighted Magnetic Resonance Images in Prostate Cancer Patients

No-reference image quality assessment method of the T2-weighted magnetic resonance images in prostate cancer patients introduced by Masoudi et al. [54] is a classification method to determine non-diagnostic images with an artifact, diagnostic images with substantial noise or motion, and diagnostic images with trivial noise or motion. The model assumes the extension of NR-IQA scans to FR-IQA, after an improvement of image quality by using CycleGAN. In the method, original images are compared with synthetic reference images. The poor quality images receives further correction from CycleGAN [54].

### 3.4. Two-Stage Multi-Modal MR Images Fusion Method Based on Parametric Logarithmic Image Processing Model

In the two-stage multi-modal MR images fusion method based on parametric logarithmic image processing model, image fusion is used to obtain a more accurate, final image [42]. It is a two-step process and uses decomposition based on stationary folk transformation (SWT) along with principal component analysis (PCA). The first and second fusion coefficients are combined using the HVS-based parameterized logarithmic image processing (PLIP) operator. To increase the accuracy, the obtained results are compared using different measures.

### 3.5. Hierarchical Non-Local Residual Networks for Image Quality Assessment of Pediatric Diffusion MRI with Limited and Noisy Annotations

The hierarchical non-local residual networks for image quality assessment of pediatric diffusion MRI with limited and noisy annotations is a deep-learning method based on local residual networks [43]. It consists of three stages, namely those involving the use of a slice-wise QA network (i.e., SQA-Net); the extracted slice features with the volume-wise QA network; and a compilation of the IQA results using the decision rule. Through these actions, this method makes the evaluation results available at different levels: namely those of slice, volume, and subject. SQA-Net is also constructed by implementing depthwise separable convolutions (DSConv) and non-local mean operation. To increase the effectiveness of the approach when working with a small amount of labeled data, semi-supervised learning and the subsequent slice with volume self-training are used.

### 3.6. HyS-Net

The spatial-related hyper-network-based MRI BIQA works on the MRIQC open dataset are based on the development of a hyper network that adapts to the content [45]. The structure of the 3D network was designed to explore spatial information of 3D images and improve the BIQA performance. In addition to relying on a hyper set that generates dynamic parameters, the method includes the extraction of spatial features and a combined network quality predictor.

### 3.7. QEMDIM

The quality evaluation using multi-directional filters for MRI (QEMDIM) is a method that enables the detection of distortions with different characteristics, e.g., Gaussian noise, motion artifacts, streaks, or aliasing [48]. It can be used not only in assessing the quality of medical images but also in assessing the performance of MR hardware and software. It is based on the feature difference between test and pre-scanned images. The performance of the method varies according to the slice position.

### 3.8. AQASB

The automatic quality assessment in structural brain magnetic resonance imaging (AQASB) is a method that focuses on the background area of the MRI image (i.e., the air) [60]. It specializes in analyzing images with distortions such as ghosting, motion, flow and wrap-around. The method consists of three steps: (1) background air region delineation; (2) computation of a model-free quality index; and (3) the computation of an additional quality index. It is developed to inform the operator about the poor quality of the measurement and to notify the need to perform an additional scan. The method has its limitations as it assumes that each scanned image has a sufficiently large percentage of background voxels to successfully perform the measurement. The method takes a limited number of artifacts into consideration. It aims to overcome the challenge of not having access to data labels and a reduction in computational time.

### 3.9. Multi-Class Cardiovascular Magnetic Resonance Image Quality Assessment Using Unsupervised Domain Adaptation

The multi-class cardiovascular magnetic resonance image quality assessment using unsupervised domain adaptation is a deep learning model for automatic cardiovascular magnetic resonance (CMR) IQA [49,61]. In the process of image quality assessment method evaluation, the distortions in the spatial domain of 2D sliced CMR images are identified. The domain adaptation is based on the trained model that is used to test the new dataset. Before the image reconstruction, the frequency domain was described to use the method on the data of k-space.

### 3.10. MRIQC

The MRI quality control tool (MRIQC) extracts quality measures and uses them as input to a binary classifier [63]. The classification is performed on the basis of binary labels from a set of MRI images. The model includes a selection of hyper-parameters in non-nested cross-validation, the training process on ABIDE dataset, evaluation on the held-out dataset, the normalization of features, and the elimination of features based on the site prediction. The release of an MRI quality control tool, MRIQC, leads to the extraction of a vector of 64 image quality measures (IQMs). The IQMs can be grouped into four categories—(1) measures based on noise measurements: the coefficient of joint variation of GM and WM (CJV), the contrast-to-noise ratio (CNR), the signal-to-noise ratio calculation (SNR) and the second quality index (QI2); (2) measures based on information theory: the entropy-focus criterion (EFC), the foreground-background energy ratio (FBER); (3) measures targeting specific artifacts: the bias field extracted estimated by the INU correction, the first quality index (QI1), the white-matter to maximum intensity ratio is the median intensity within the WM mask (WM2MAX); (4) other measures: the full-width half-maximum (FWHM), estimation of the ICVs, the residual partial volume effect feature (rPVE), several summary statistics such as the mean or standard deviation (SSTATs), and overlap of tissue probability maps (TPMs).

### 3.11. Brain and Cardiac MRI Images in Multi-Center Clinical Trials

The method assumes that MRI slices possess statistical properties describing different levels of contrast degradation [65]. Thus, to each level of contrast-distorted MRI slice, a set of pixel configurations is assigned. The IQA process is divided into four steps. Firstly, local contrast features are extracted from the test image, then the mean and standard deviation are computed. To obtain two separate z-scores, the mean and standard deviation are processed. As a result, the prediction of the contrast quality score, and the texture contrast quality score is performed. Focused on the labeling problem and the central limit theorem, the approach aims to describe each possible level of contrast degradation in an MRI slice. It perceives images with artifacts as darker than denoised ones, which also have lower contrast. The method predicts higher texture contrast quality score.

### 3.12. Modified Blind/Referenceless Image Spatial Quality Evaluator (BRISQUE)

Modified blind/referenceless image spatial quality evaluator (BRISQUE) [47] is a model initially created with natural images in mind, but it was adapted to evaluate the quality of MR images [46]. It uses locally normalized luminosity coefficients for calculations, the MR image function and the difference mean opinion score (DMOS) for training. The goal of its development was to create a method that would be useful when working on images with all types of distortions.

### 3.13. R50GR18

This method presents a different approach to image quality assessment as it is based on the fusion of neural networks (ResNet50, GoogLeNet, and ResNet18) which then take part in the transfer-learning process [50]. The large diversity of the architectures selected for the study and fusion allows the assessment of a large spectrum of distortions. The method uses support vector regression (SVR) [67] on features extracted from connected networks to improve the ability to evaluate the quality of images. Additionally, a network modification is applied, in which the last three layers of each network used are replaced with a fully connected layer and the regression layer to perform the regression task. Moreover, the size of the input image is not constant but is adapted to the size of the input network.

### 3.14. Entropy-Based Magnetic Resonance Image Quality Assessment Measure (ENMIQA)

In the method, the entropy of the extrema of local intensity differences representing filtered versions of an input image is used for quality prediction [51]. Specifically, the quality prediction is expressed by the entropy of a sequence of extrema numbers obtained with the thresholded non-maximum suppression (NMS) applied to filtered MR images.

### 3.15. No-Reference Image Quality Assessment of Magnetic Resonance Images with High-Boost Filtering and Local Features (NOMRIQA)

The method introduced by Oszust et al. [52], NOMRIQA, uses high-boost filtering to amplify high-frequency points allowing for the effective detection of distortions. Detected interest points in filtered images are described using the fast retina key-point (FREAK) descriptor and then represented by a histogram of such descriptors. The method builds a quality model with the SVR approach.

#### 3.15.1. PSNR/SNR

The peak signal-to-noise ratio (PSNR) in numerous studies is used as an NR-IQA tool. For this reason, in this paper, PSNR and signal-to-noise ratio (SNR) [68,69,70,71] is also described. PSNR depends on the value of RMSE among the target image and the reference image. It is calculated as:

(1) $$\mathrm{PSNR}=10log\frac{m^{2}max}{RMSE^{2}},$$

where $m_{max}$ means the maximum pixel score. There is a poor correlation of PSNR with subjective quality assessment performed by human observers. However, this dissimilarity can be captured in each type of distortion by the perceptual complexity of the target image. There have been attempts to improve the PSNR performance by the use of a linear score mapping process with the use of factors such as image-free energy and type of distortion.

#### 3.15.2. Maximum Difference

The maximum difference (MD) presents the maximum of the error signal [72]. Additionally, the quality of an image decreases with the growth of the MD value.

(2) $$\mathrm{MD}=Max(|C_{ef}-D_{ef}|),$$

where e=1,2,…,n,f=1,2,…,m.

#### 3.15.3. Normalized Cross-Correlation

The normalized cross-correlation (NCC or NK) measures the similarity of the image sets and detects patterns or the object of an image [72,73]. The metric is used in image registration.

(3) $$\mathrm{NK}=\frac{\sum_{j=1}^{n}\sum_{i=1}^{m}z\left(j,i\right)Zs\left(j,i\right)}{\sum_{j=1}^{n}\sum_{i=1}^{m}{\left(z\left(j,i\right)\right)}^{2}},$$

where *n* denotes the number of pixels in the horizontal direction; *m* denotes the number of pixels in vertical direction; s(j,i) denotes the filtered image at *j* and *i* coordinates; and z(j,i) is the noisy image at *j* and *i* coordinates.

## 4. Evaluation of IQA Models

The development of image quality assessment methods is stimulated by the existence of suitable databases and widely accepted protocols, ensuring that the methods are fairly and thoroughly compared.

### 4.1. Databases

**Figure 2:**
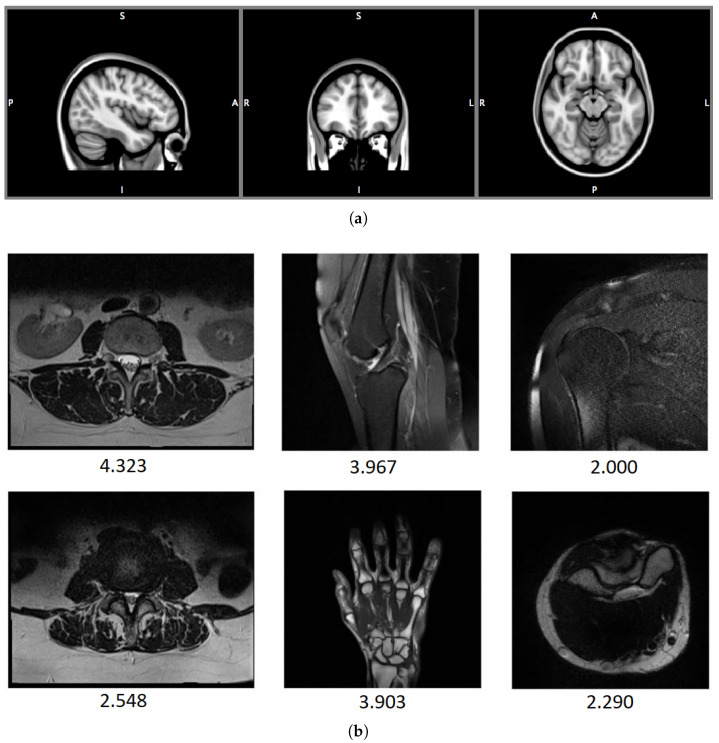
Exemplary MR images from OpenfMRI (**a**) and DB1 (**b**) databases. MOS values for images from DB1 database are also displayed.

There are many medical databases created to develop and test methods related to MR. Most of them are assessed by authors of approaches to provide subjective scores for training the methods. Only rare examples contain images along with human scores. Below, the most commonly used databases are presented. Their summary and exemplary images are shown in Table 2 and Figure 2, respectively.

#### 4.1.1. OpenfMRI

The OpenfMR collection contains the MR and EEG of human brain images [64]. It accepts all forms of data that include MR imaging, is perceived as simple in the organization, and has no data access limitations. The data usually consist of four-dimensional datasets. However, depending on the number and length of scanning runs, spatial resolution, and the number of slices acquired, an fMRI study can range from fifteen patients for most studies. The project was created to provide the open sharing of neuroimaging resources.

#### 4.1.2. ADNI

The Alzheimer’s Disease Neuroimaging Initiative (ADNI) was developed to contribute to the early detection and tracking of Alzheimer’s disease [58]. It was founded in 2004 and is divided into four areas of study: ADNI-1, ADNI-GO, ADNI-2, and ADNI-3. ADNI-1 includes 800 patients and uses MRI and PET imaging measures. ADNI-GO consists of 1000 patients and its main purpose for evaluation is the examination of biomarkers in the earlier stages of Alzheimer’s disease. The ADNI-2 and -3 studies expanded upon previously acquired image databases with hundreds of new examples. A new cohort was added, significant memory concern (SMC), in addition to brain scans that detect tau protein tangles (tau PET). In recent years, ADNI-3 has gathered data from scientists at 59 research centers in the United States and Canada.

#### 4.1.3. National Resource for Quantitative Functional MRI

The National Resource for Quantitative Functional MRI project was created to design quantitative magnetic resonance acquisition and processing technology to track brain changes during neurodevelopment or neurodegeneration. This technology is developed in collaboration with a large community of specialists from several institutions in the USA. The overall scope is divided into four databases of MR images (D1–4). The D1 database concerns metabolic (S)I and operates on high-resolution MR spectroscopic imaging (MRSI) [58,74] in the research of brain and spine metabolism at the magnetic field strengths of 3 and 7 Tesla. The D2 database is related to psychological MRI that aims to discover tissue biomarkers to provide early information about physiological and metabolic changes in clinical imaging. The D3 database is devoted to functional MRI that uses information about the blood oxygenation level to assess changes in the brain that can be the cause of many diseases such as autism, ADHD, or Alzheimer’s [75]. The objective of the last database, D4, is to develop image analysis technologies able to integrate different anatomical representations of the brain based on multi-modal MRI, including multi-contrast anatomical MRI, functional MRI (fMRI), and MR spectroscopy (MRS) into a common framework [76].

#### 4.1.4. Autism Brain Imaging Data Exchange (ABIDE)

ABIDE is a collection of 16 worldwide imaging sites that are openly sharing 1112 datasets composed of structural and resting-state pre-processed MRI [59,77]. The age of the individuals under control varies from 7 to 64 years old. The pre-processing was performed using: the Connectome Computation System (CCS), the Configurable Pipeline for the Analysis of Connectomes (CPAC), the Data Processing Assistant for Resting-State fMRI (DPARSF) and the Neuroimaging Analysis Kit. Four pre-processing strategies were performed with each pipeline: all combinations of with and without filtering and with and without global signal correction as well as the statistical derivatives for each pipeline and strategy were calculated by the CPAC software. Three different pipelines were used: ANTS, CIVET and Free Surfer.

#### 4.1.5. 1.5 T T2-Weighted MR Image Databases: DB1, DB2

The DB1 [52] and DB2 [51] datasets contain 70 and 240 MR images, respectively. The DB1 benchmark consists of images selected from 1.5 T MR T2-weighted sagittal sequences of several body locations, i.e., knee, shoulder, or spine. The resolution of images in the dataset is between 192 × 320 and 512 × 512. Images were taken under different conditions affecting the image quality (IPAT software to make generalized autocalibrating partially parallel acquisitions (GRAPPAs); GRAPPA3 [78]). The DB1 also contains the mean opinion score (MOS) ranging from 1 to 5 which was obtained in tests with a large group of radiologists. The greater MOS value denotes the better quality of the image. The DB2 collection contains T2-weighted MR images acquired during routine diagnostic exams of different body locations, including shoulders, knees, or cervical and lumbar spine. The dataset contains images of resolution from 192 × 320 to 512 × 512 and MOS (1–5). To acquire images of different quality in a controlled way, the parallel imaging technique was applied. Hence, a group of radiologists was also invited for the assessment of the image quality.

### 4.2. Evaluation Protocol

To compare the performance of regression-based IQA approaches, four performance criteria are typically used: Spearman rank-order correlation coefficient (SRCC); Kendall rank-order correlation coefficient (KRCC); and Pearson linear correlation coefficient (PLCC); and root mean square error (RMSE). The higher the SRCC, KRCC and PLCC, and the lower the RMSE, the better the output of the IQA method is [79,80,81]. The works on IQA methods that classify images often report the accuracy measured as the number of correctly classified images, receiver operating characteristic curves (ROC), or the area under the curve, showing the relation of the performance and a threshold. Furthermore, in the methods that classify images, the quality assessment is commonly formulated by the use of diagnostic and non-diagnostic labels from a trained observer [82,83,84]. The train–test split dataset protocol is used to calculate the performance of algorithms in cases they are used to make predictions on data not used to train the model. The results allow for comparing the performance of machine learning algorithms for chosen predictive modeling problems. Typically, IQA databases are randomly divided into a training set with 80% distorted images, and the remaining 20% of distorted images are used to test the model [85]. The division is then repeated and the metric of the quality measure is reported. For images with artificially created distortions, the images compatible with the same reference image are paired with the same set to provide the total separation of the training and testing content.

PLCC is used for prediction accuracy between the sets of values. It is calculated as follows:

(4) $$\mathrm{PLCC}=\frac{{{\bar{E}}^{O}}^{T}{\bar{O}}^{s}}{\sqrt{{{\bar{E}}^{O}}^{T}{\bar{E}}^{O}{{\bar{O}}^{s}}^{T}{\bar{O}}^{s}}},$$

where $\bar{E^{o}}$ and $\bar{O^{s}}$ are mean-removed vectors. RMSE is obtained as:

(5) $$\mathrm{RMSE}=\sqrt{\frac{{\left(E^{o}-O^{s}\right)}^{T}\left(E^{o}-O^{s}\right)}{n}},$$

where *n* means the total number of images. SRCC evaluates the prediction of monotonicity and is calculated as follows:

(6) $$\mathrm{SRCC}=1-\frac{6\sum_{i=1}^{n}b_{i}^{2}}{n(n^{2}-1)},$$

where $b_{i}$ means the difference between the *i*-th image in $E^{o}$ and $O^{s}$, i=1,2,…,n. Finally, KRCC is obtained as:

(7) $$\mathrm{KRCC}=\frac{n_{c}-n_{d}}{0.5n(n-1)},$$

where $n_{c}$ is the number of concordant pairs in the dataset, and $n_{d}$ means the number of discordant pairs. Since the calculation of PLCC and RMSE involves the nonlinear mapping of objective scores $e^{o}$ into subjective opinions $O^{s}$, the mapping model can be represented by:

(8) $$e^{0}=y_{1}\left(\frac{1}{2}-\frac{1}{1+exp(y_{2}\left(e^{0}-y_{e}\right))}\right)+y_{4}e^{0}+y_{5},$$

where $y=[y_{1},y_{2},\ldots ,y_{5}]$.

There are three error measurement metrics commonly used in model evaluation: RMSE [80], mean square error (MSE) [86] and mean absolute error (MAE) [87]. The metrics calculate the objective quality scores after regression, *O*, and the errors between the datasets, *E*:

(9) $$\mathrm{MAE}\left(O,E\right)=\frac{1}{n}sum_{i=1}^{n}\left|O_{i}-E_{i}\right|,$$

(10) $$\mathrm{MSE}\left(O,E\right)=\frac{1}{n}sum_{i=1}^{n}{\left(O_{i}-E_{i}\right)}^{2},$$

where *n* denotes the size of the dataset.

## 5. Conclusions

In this paper, diverse approaches to the automatic NR quality assessment of MR images were presented, and detailed characteristics of recent methods, used features, and image benchmarks that stimulate the development in this field were provided. As presented, the number of works devoted to the assessment of MR images is relatively low, despite their usability in practice. First, such approaches adapt methods from the IQA of natural images or perform image processing steps that address the characteristics of MR images. More recent methods often use or introduce powerful deep learning architectures. Despite the present division of the methods into quality prediction and quality classification methods, both types aim to indicate which images should not be used for diagnostic purposes. However, the methods based on regression can be used for the development of image enhancement algorithms due to the capability of distinguishing small quality differences instead of binary classifiers that only reject unsuitable images.

Approaches are also divided into methods that assess slices and methods that can be employed for 3D quality evaluation. However, the development of both types of techniques requires access to large-scale MR image databases that also contain subjective scores obtained in tests with human participants. Nowadays, the creation of such databases can be considered the most challenging problem in MR-IQA. Its solution would lead to the emergence of more accurate quality prediction approaches.

## Figures and Tables

**Table 1:** Comparison of NR-IQA methods in terms of employed techniques, features, and used datasets.

Method	Approach and Features	Number of Features	Datasets
A two-step automated quality assessment for liver MR images based on convolutional neural network [53]	Patch-based strategy CNN in image region segmentation ROI	-	Not defined in the paper
Semi-supervised learning for fetal brain MRI quality assessment with ROI consistency [7]	Semi-supervised learning ROI consistency Mean teacher model	-	Scans acquired at Boston Children’s Hospital
No-reference image quality assessment of T2-weighted magnetic resonance images in prostate cancer patients [54]	Generative adversarial network Weakly supervised Trained deep classifier	-	National Cancer Institute (NCI) PIE-AAPM-NCI Prostate MR Gleason Grade Group Challenge NIH Clinical Center
Two-stage multi-modal MR images fusion method based on parametric logarithmic image processing (PLIP) model [55]	Two-stage MRI fusion PCA and PLIP operators Stationary wavelet transform	-	Whole Brain Atlas [56]
Hierarchical non-local residual networks for image quality assessment of pediatric diffusion MRI with limited and noisy annotations [43]	Slice-wise, volume-wise, and subject-wise IQA Non-local residual networks Semi-supervised learning	-	Database from the Center for Magnetic Resonance Research (CMRR) at the University of Minnesota
HyS-net [45]	Content-adaptive hyper-network A spatial feature extraction Network-based quality predictor	-	Open dataset, MRIQC [57]
QEMDIM [48]	Difference of statistical features between test images MSCN coefficients Multi-directional filtered coefficients (MDFC)	20	ADNI [58] ABIDE [59]
AQASB [60]	Background-connected distortions Decent level of background voxels	-	ADNI [58]
Multi-class cardiovascular magnetic resonance image quality assessment using unsupervised domain adaptation [49,61]	Unsupervised domain adaptation Spatial and frequency domains K-space manipulation	512	UK Biobank Cardiac MRI dataset, York University [62] K-space manipulation
MRIQC [63]	Quality measures Binary classifier	64	ABIDE [59] OpenfMRI [64]
Brain and cardiac MRI images in multi-center clinical trials [65]	The moments-preserving property application Measures the differences in texture contrast	The number of features depends on the image	NeuroRx research Inc. BrainCare Oy ADNI [58] Department of Diagnostic Imaging of the Hospital for Sick Children in Toronto
Modified-BRISQUE [46]	Luminosity, image characteristics NSS	36	Sirix DICOM Viewer MRI database MR images from the Academy Unit of Radiology, University of Sheffield
R50GR18 [50]	Fusion of deep network architectures SVR	3584	DB1 [52] DB2 benchmarks [51]
ENMIQA [51]	Thresholded local intensity differences obtained by using the non-maximum suppression (NMS) operation Entropy of a sequence of extrema numbers	1	DB1 [52]
NOMRIQA [52]	FAST features Histograms of binary descriptors	3840	Simulated Brain Database (SBD) [66] DB1 [52]

**Table 2:** Details of the MR image datasets.

Name	Year	No. of Images	Link (Accessed on 27 April 2022)
OpenfMRI	2010	Not specified/repository of datasets	openfmri.org
ADNI-1	2004–2010	200 elderly controls, 400 MCI, 200 AD	adni.loni.usc.edu
ADNI-GO	2009–2011	Existing ADNI-1 + 200 early MCI	adni.loni.usc.edu
ADNI-2	2011–2017	Existing ADNI-1 and ADNI-GO + additional images	adni.loni.usc.edu
ADNI-3	2017–2022	Existing ADNI-1, ADNI-GO, ADNI-2 + additional images	adni.loni.usc.edu
ABIDE I	2012	1112 datasets	fcon1000.projects.nitrc.org
ABIDE II	2016	Existing ABIDE I and 1000 datasets	fcon1000.projects.nitrc.org
DB1	2020	70	marosz.kia.prz.edu.pl/ENMIQA.html
DB2	2020	240	marosz.kia.prz.edu.pl/NOMRIQA.html

## Data Availability

No new data were created or analyzed in this study. Data sharing is not applicable to this article.
